# Is external hydrocephalus a possible differential diagnosis when child abuse is suspected?

**DOI:** 10.1007/s00701-021-04786-3

**Published:** 2021-03-12

**Authors:** Joseph Scheller, Knut Wester

**Affiliations:** 1 Neurologist in Private Practice, 600 Reisterstown Rd #301, Baltimore, MD 21208 USA; 2 Department of Clinical Medicine K1, University of Bergen, N 5021 Bergen, Norway

**Keywords:** Abusive head injury, AHT, Benign external hydrocephalus, Child abuse, SBS, Shaken baby syndrome, Subdural haematoma, subdural hygroma

## Abstract

**Background:**

Criteria for diagnosing abusive head trauma (AHT) or “shaken baby syndrome” are not well defined; consequently, these conditions might be diagnosed on failing premises.

**Methods:**

The authors have collected a total of 28 infants, from the US (20) and Norway (8), suspected of having been violently shaken, and their caregivers had been suspected, investigated, prosecuted or convicted of having performed this action. Among 26 symptomatic infants, there were 18 boys (69%) and 8 girls (31%)—mean age 5.1 month, without age difference between genders.

**Results:**

Twenty-one of 26 symptomatic children (81%) had a head circumference at or above the 90 percentile, and 18 had a head circumference at or above the 97 percentile. After macrocephaly, seizure was the most frequent initial symptom in 13 (50%) of the symptomatic infants. Seventeen (65%) of the symptomatic infants had bilateral retinal haemorrhages, and two had unilateral retinal haemorrhages. All infants had neuroimaging compatible with chronic subdural haematomas/hygromas as well as radiological characteristics compatible with benign external hydrocephalus (BEH).

**Conclusions:**

BEH with subdural haematomas/hygromas in infants may sometimes be misdiagnosed as abusive head trauma. Based on the authors’ experience and findings of the study, the following measures are suggested to avoid this diagnostic pitfall: medical experts in infant abuse cases should be trained in recognising clinical and radiological BEH features, clinicians with neuro-paediatric experience should always be included in the expert teams and reliable information about the head circumference development from birth should always be available.

## Introduction

Child abuse has been recognised as an important paediatric problem for the last half century. Among different forms of child abuse, injuries to the central nervous system have received particular attention.

In 1971, the British neurosurgeon Norman Guthkelch published an article on subdural haematomas (SDH) in infants with the title *Infantile Subdural Haematoma and Its Relationship to Whiplash Injuries* [25]. His patient material consisted of 23 children suspected of suffering from “battered child syndrome”. Thirteen of these had subdural haematoma, but in two of these infants—both six-month-old boys—there was no external signs of trauma, which caused Guthkelch to believe that “*there was very strong reason indeed to suppose that the mechanism of production of the subdural haemorrhage had been by shaking rather than battering*”.

The notion that violent shaking alone could cause SDH was soon adopted by others, such as by Caffey already in 1972 [9], who in 1974 renamed the condition “*Whiplash Shaken Infant Syndrome*” [10]. Caffey emphasised how difficult it was to find support for the hypothesis from observations or admissions: “*Direct evidence of trauma through admission by the parent-assailant or the statement of a witness is rarely obtained or obtainable*” [10]. The term “*Shaken Baby Syndrome*” (later often referred to as SBS) was introduced by Ludwig and Warman [40] in 1984 in a review of 20 infants and young toddlers that allegedly had been injured by shaking, although shaking had not been witnessed.

A “triad” of findings, comprising SDH, retinal haemorrhages (RH) and signs of brain injury, has gradually emerged and been accepted as a strong indicator that shaking indeed has taken place and that it also is the *cause* of the triad [11, 14].

Nearly half a century after Guthkelch suggested that shaking could cause SDH, it is difficult to find scientific support for this hypothesis; two extensive literature reviews have failed to locate scientific contributions of high scientific quality [13, 15, 63]. These reviews question the validity of the triad as an evidence of violent shaking has been questioned, mainly because of circular reasoning.

Usually, conviction for child abuse requires supporting evidence. A recent German survey of 68 accused cases revealed conviction for child abuse in 19; 15 of which included confessions by the alleged perpetrators, but four without [16]. Still, recent surveys and court cases show that the triad may be accepted as an unequivocal sign of child abuse and lead to conviction without a confession or supporting evidence [65].

The epidemiological characteristics—male preponderance and low age—are not only shared by most published SBS/AHT populations. Nearly identical epidemiological features have been noted for the neuro-paediatric condition “*Benign/idiopathic external hydrocephalus* (*BEH*)”, a condition that is also known as “*Benign enlargement of the subarachnoid spaces* (*BESS*)”, and “*Benign familial macrocephaly*” reviewed in [77].

BEH develops during infancy; most of these children are born with a close-to-normal head circumference (HC) that increases rapidly during the first months of life, and there is a marked male preponderance as well [72, 76].

BEH appears to share epidemiological and radiological characteristics with SBS/AHT. A combination of clinical and radiological findings in BEH includes macrocephaly, normal or moderately enlarged lateral ventricles and increased extra-axial fluid [2, 17, 20, 38, 43], in addition to specifically defined intracranial distances, such as craniocortical (CCW), sinocortical (SCW) widths and interhemispheric distance (IHD), for details, see [72]. This extra-axial fluid, which possibly is a reminiscent of birth-related SDH/subdural hygroma (SDHy), is usually *chronic* with small volumes of fresh blood, a key feature in the radiological diagnosis of SBS/AHT, but also a common complication to BEH. [4, 21, 23, 26, 31, 34, 35, 44, 45, 47, 48, 52, 55].

Several authors have pointed to the risk that a spontaneously occurring SDH in infants with BEH can be misdiagnosed as SBS/AHT [26, 55, 69].

The similarities between the findings in SBS/AHT and BEH and the uncertainty these similarities create are alarming. We acknowledge that questioning the diagnostic safety in child abuse may endanger children at risk for abuse. This risk has to be balanced, if at all possible, against the risk of wrongly accusing caregivers and thus destroying innocent families. Such a diagnosis must be made with the utmost care to avoid mistakes in either direction. Still, the triad has been regarded as important for indicating need for further investigation. To minimise mistakes, we hypothesise that BEH can be recognised as a differential diagnosis to SBS/AHT in infants where the triad or its components have been diagnosed—and that this specific combination of findings needs recognition to avoid diagnostic pitfalls.

The present study describes and analyses a group of infants that were diagnosed as having been violently shaken, but where clinical and radiological features were more compatible with BEH complicated by a chronic subdural haematoma (CSDH) or hygroma than with SBS/AHT. Our aim is to raise awareness about this possible pitfall in the diagnosis of AHT/SBS, and above all, to reveal factors that may ease the differentiation between abuse and BEH and thus improve the diagnostic safety.

## Material and methods

### The patients

The authors have collected 28 consecutive case histories after being contacted as expert witnesses by 26 families with a total of 28 (20 from the US seen by JS; 8 from Norway seen by KW) infants that were suspected of having been violently shaken, and their caregivers had been accused or convicted of having performed this shaking. The suspicion or diagnosis of child battering was exclusively based on the radiological imaging, but later followed up by ophthalmological examinations for retinal haemorrhages.

All included US infants had been diagnosed as SBS/AHT by a “Child Abuse Physician” (CAP), which are paediatricians who have done a fellowship in child abuse. The ones with seizures were also seen by a paediatric neurologist, who relied on the CAP diagnosis. The Norwegian infants had been diagnosed as SBS/AHT by specialists in forensic medicine (*n*=3), pathology (*n*=2) and/or paediatrics (*n*=3). In this country, only a few experts are involved in all such court cases. All included infants had undergone ophthalmological exam, blood tests to rule out a coagulopathy and brain and skeletal imaging.

For each child, we had access to all medical documentation, including neuroimaging and the complete medical records. We analysed the medical records and neuroimaging for epidemiological features and symptoms and clinical and radiological findings that might be compatible with BEH. Specifically, we recorded macrocephaly as defined by measurement of head circumference, normal or moderately enlarged ventricles and extra-axial fluid. Three distances were used to quantify extra-axial fluid: craniocortical (CCW) and sinocortical (SCW) widths and interhemispheric distance (IHD). No validated normal values exist for these distances in the literature; the upper limit above which the CCW is likely to be abnormal, ranges from 4 to 10 mm, for SCW 2–10 mm and for IHD 6–8.5 mm, see [72].

Macrocephaly was defined as a head circumference >95th percentile for age. Subdural hygroma was defined as extra-axial fluid outside the arachnoid membrane that was less dark on T1 and FLAIR images than adjacent CSF and SCW, CCW and IHD within the ranges given above.

Two three-month-old infant girls (#23, 25) were asymptomatic, but were nevertheless included; they had both been examined and diagnosed as SBS/AHT because their symptomatic twin brothers had been diagnosed as shaken.

The caregivers of all children gave their consent to publish the information included in the present article.

## Results

For a brief overview of epidemiological, radiological and clinical details, see Table 1. All the children had fluid collections that were described as chronic subdural haematomas, and none of the infants had evidence of direct impact to the head or signs of any other external trauma, such as rib fracture, limb fracture or neck injury.

**Table 1:** Epidemiological, clinical and radiological details. *ASDH* acute subdural haematoma, *CSDH* chronic subdural hygroma, *RH* retinal haemorrhage. As described in the main text, pats. #23 and #25 were asymptomatic twin sisters of pats #22 and #24. In all patients with ASDH, the volume of acute blood represented only a minor fraction of the extra-cerebral fluid

Pat. #	Sex	Age at first symptom (months)	Initial symptoms	HC	MRI/CT findings	Age (months) at neuroimaging	RH	Comments
1	M	3	Vomiting, seizure	98%	CSDH, ASDH, SAH	4	None	
2	M	5	Seizure	77%	CSDH, ASDH	6	Bilat.	
3	M	6	Seizure	98%	CSDH	6	Bilat.	
4	M	2	Macrocephaly	99%	CSDH	2	Bilat.	NS drainage, motor oil fluid, membranes
5	F	10	Lethargy	93%	CSDH, ASDH	10	Bilat.	
6	M	5	Macrocephaly	99%	CSDH, BESS	5	None	NS drainage, hygroma
7	M	1	Seizure	95%	CSDH, ASDH, BESS	1	Bilat.	
8	F	6	Macrocephaly	-	CSDH, ASDH	6	Bilat.	
9	M	7	Seizure	99%	CSDH, ASDH	7	None	
10	M	3	Vomiting, seizure	99%	CSDH, ASDH	3	Bilat.	
11	M	4	Vomiting	99%	CSDH, BESS	4	None	
12	F	2	Seizure	-	CSDH, ASDH	2	Bilat.	
13	M	5	Seizure, rapid HC growth	35%	CSDH, ASDH	5	Unilat.	
14	F	3	Vomiting, 6^th^ nerve palsy	99%	CSDH	5	None	NS drainage
15	M	5	Possible seizure	99%	CSDH, ASDH	5	Bilat.	NS drainage
16	M	8	Seizure	99%	CSDH, ASDH	8	Bilat.	
17	M	2	Vomiting, irritable	99%	CSDH, ASDH	2	Bilat.	
18	M	5	Lethargy, vomiting	85%	CSDH, ASDH	5	None	
19	F	4	Irritable	99%	CSDH, ASDH	4	Unilat.	
20	F	1	Macrocephaly, seizure	99%	CSDH, ASDH	1	None	
21	F	12	Psychomotor delay, falls, seizure	75–90%	CSDH, ASDH	12	Bilat.	
22	M	3	Seizure, respiratory arrest	2 cm above 97%	CSDH, ASDH	3	Bilat.	4 weeks premature NS drainage: yellow fluid
23	F	3	Twin sister of #22	75%	CSDH	4	None	4 weeks premature
24	M	3	Seizure	97%	CSDH, ASDH	3	Bilat.	5 weeks premature
25	F	3	Twin sister of #24	75%	CSDH	4	None	5 weeks premature
26	M	13	HC rapid increase	97%	CSDH	13	Bilat.	
27	F	2	Tense fontanel, frontal bossing, psychomotor delay	3 cm above 97%	CSDH	2	Bilat.	NS drainage, chronic haematoma
28	M	13	Psychomotor delay, possible seizures, rapid HC growth	90%	CSDH, ASDH	13	Bilat.	

### Epidemiological, clinical and radiological BEH characteristics

#### Epidemiology

Among the 26 symptomatic infants, there were 18 boys (69%) and 8 girls (31%)—mean age 5.1 months (median age 4.5, range 1–13), without any age difference between the genders.

### Head circumference (HC)

For two girls (#8 and 12), exact information about the HC was not available in the medical records. Twenty-one infants (81% of the 26 symptomatic children) had a head circumference at or above the 90 percentile, 15 (71%) boys and six girls (29%); 18 had an HC at or above the 95 percentile, and thus fulfilled the set criterion for “macrocephaly”.

### Neuroimaging

#### Craniocortical (CCW) and sinocortical (SCW) widths and interhemispheric distance (IHD)

CT and/or MRI revealed radiological characteristics compatible with BEH with craniocortical (CCW) and sinocortical (SCW) widths of 6 mm or above in all infants, and an interhemispheric distance (IHD) > 6 mm in all, except two (pats. # 3 and 12), who had an IHD of only 4 mm. The average of all measurements (CCW, SCW and IDH) was however far above 6 mm in all infants.

### Subdural haematomas (SDH) and hygromas (SDHy)

**Fig. 1:**
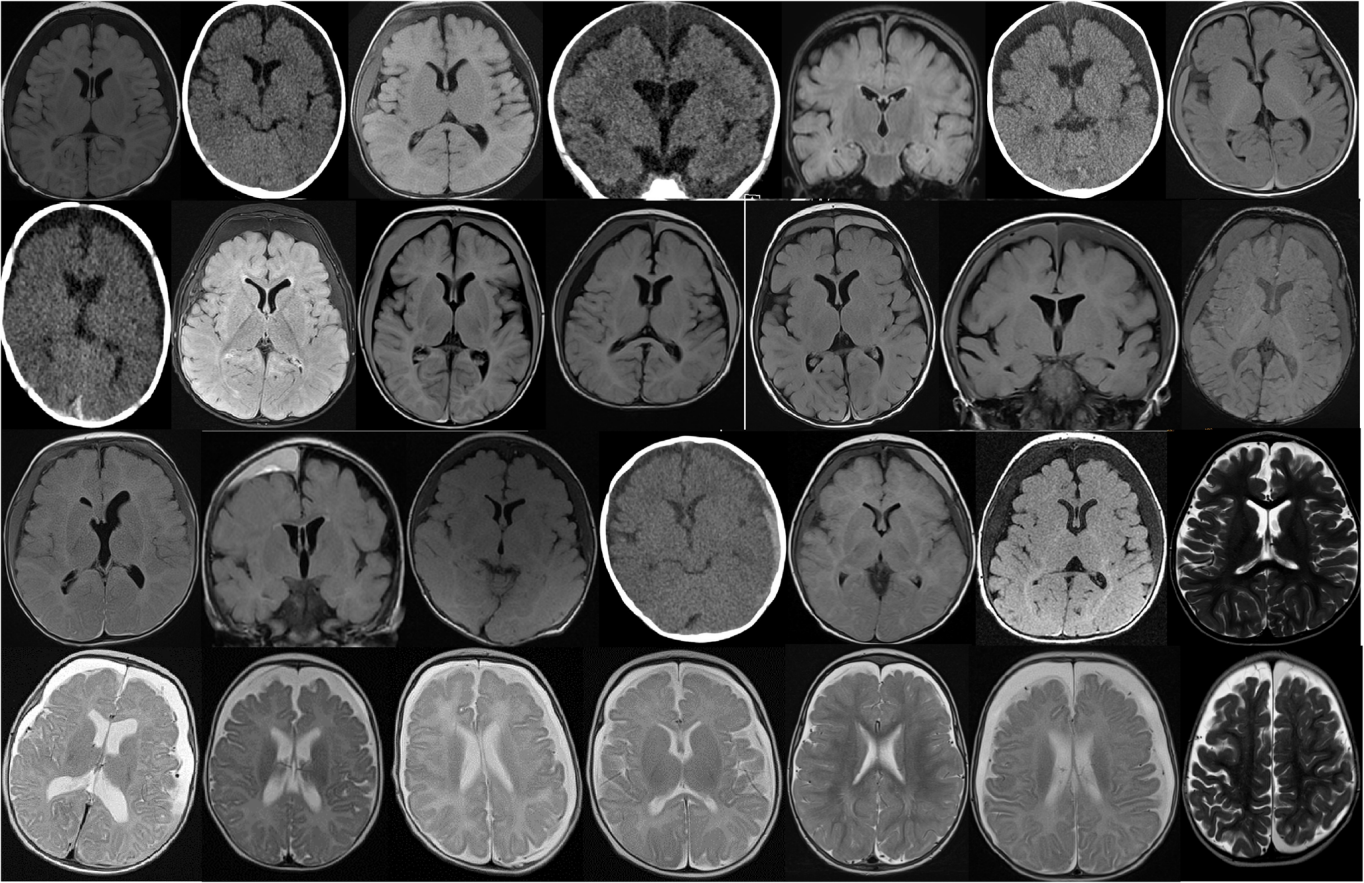
CT or MRI images approximately at the level of the foramina of Monro for 27 of the infants and one image closer to vertex for pat #28 (lower right). The patients are displayed in the following order, from left to right: upper row pats. # 1–7, second row: pats. #8–14, third row: pats. #15–21, and bottom row: pats. #22–28

**Fig. 2:**
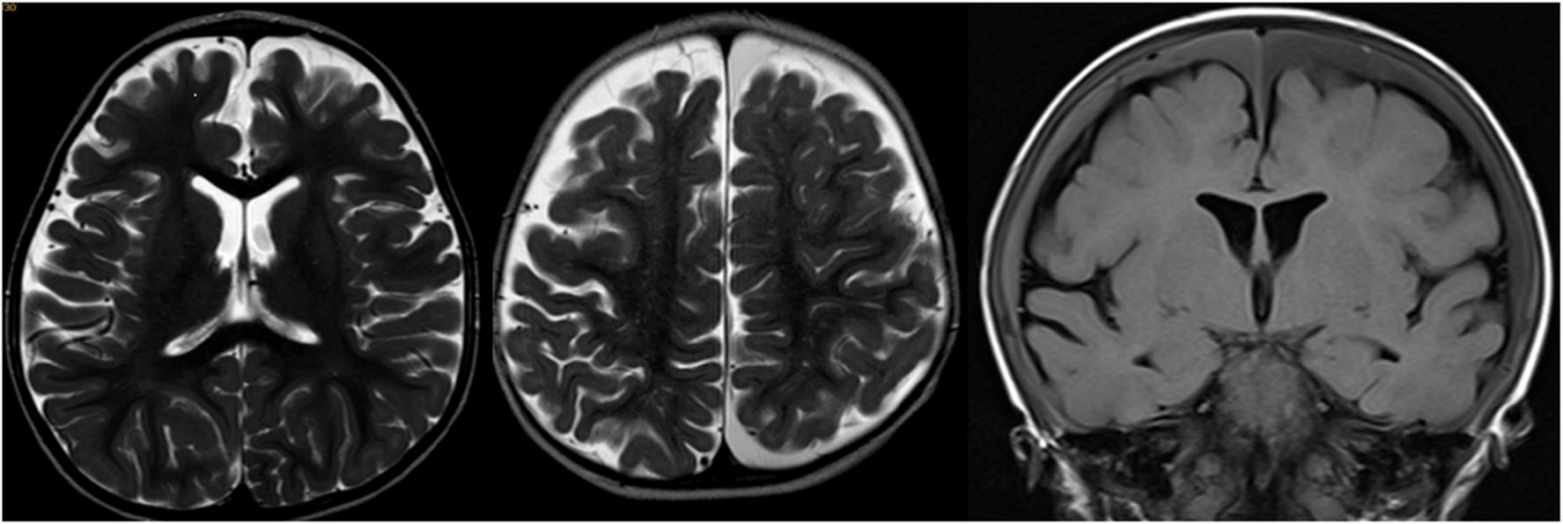
MRI images showing extra-cerebral fluid in the subarachnoid space (pat. #21—left), chronic haematomas localised in the left frontal and occipital subdural compartment (pat. #28—middle) and a globally distributed bilateral chronic SDH (pat. #13—right). Please note that the haematomas do not seem to compress the underlying cortex or the ventricles and that there is a brim of CSF between the haematomas and the cortical surface

For all infants, the neuroimaging revealed extra-cerebral fluid compatible with chronic subdural hygroma or chronic subdural haematoma (CSDH), often containing blood of different ages, including small amounts of acute and coagulated blood (acute subdural haematoma ASDH) in 19 patients, see Table 1 and Fig. 1. For 21 infants, the extra-cerebral fluid was located globally in the entire subdural compartment, either as a hygroma or as a chronic SDH/SDHy. For the remaining seven infants, the MRI showed extra-cerebral fluid with more localised subdural haematomas, for an example see Fig. 2. With only two exceptions (pats # 3 and 16), the subdural fluid collections/haematomas did not compress or flatten the cortical surface, in fact the MRIs showed a brim of normally looking CSF between the subdural fluid collection/haematoma and the apparently normal cortex in all infants, even in the two exceptions mentioned above. The lateral ventricles were of normal size or moderately enlarged, and the SDHs caused a unilateral, slight compression of one lateral ventricle in only two patients (#3 and 9). A moderate midline shift was observed in two patients (pats. # 18 and 20).

### Additional symptoms and clinical findings

#### Seizures

After macrocephaly, seizure was the most frequent initial symptom; 13 (50%) of the 26 symptomatic infants had seizure as first symptom, 10 boys and 3 girls, with a mean age of 4.5 months.

#### Retinal haemorrhages (RH)

**Fig. 3:**
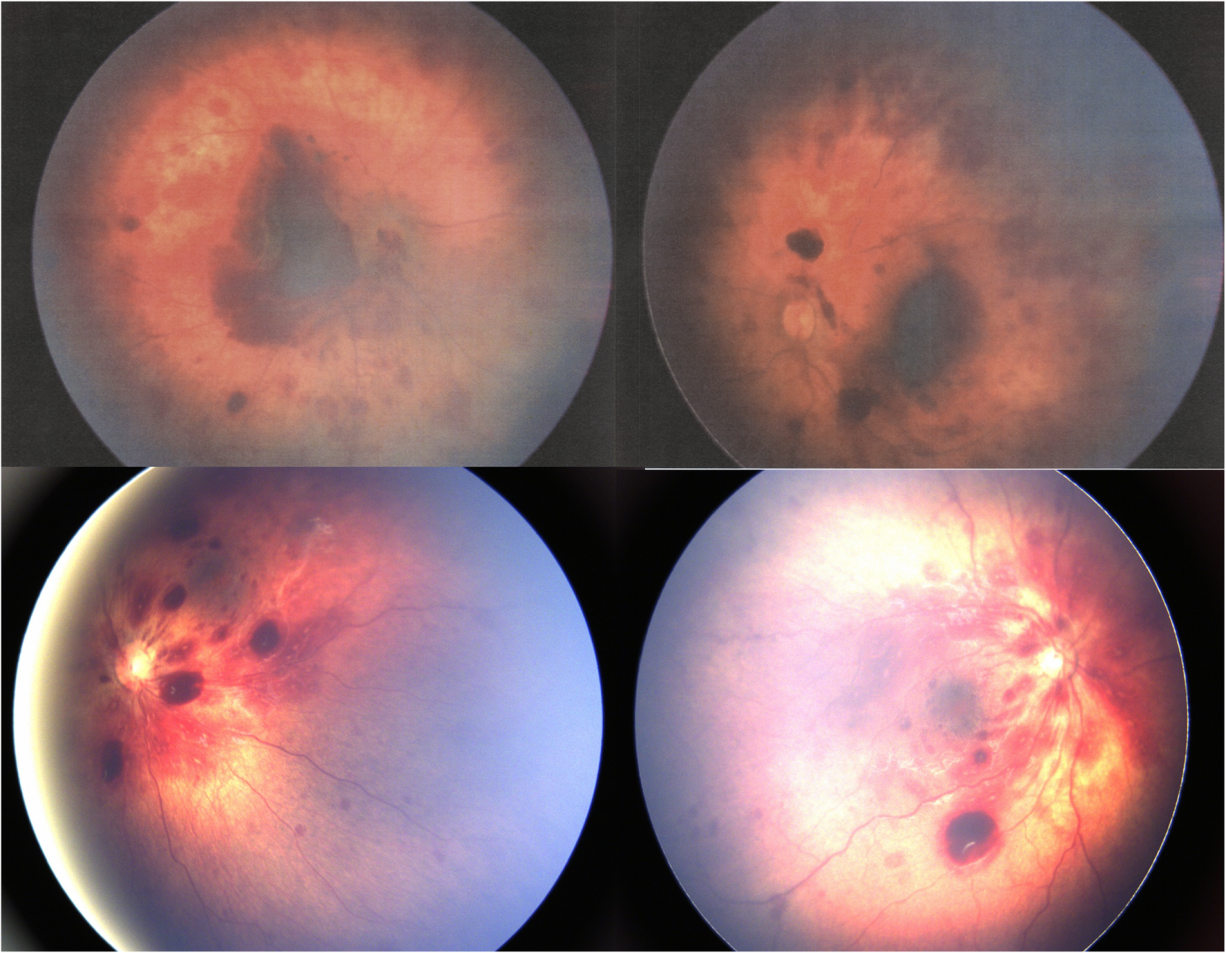
Fundoscopy images of patients #2 (upper row) and #17 (lower row), showing widespread, bilateral retinal haemorrhages. Left eye to the left, right eye to the right.

Seventeen (65%) of the symptomatic infants had bilateral RH, 12 boys and five girls, two had unilateral RH, and seven had normal fundi. The bilateral RHs had an extensive distribution and involved several retinal layers. Nine (69%) of the 13 infants with seizures also had bilateral RH, and one had a unilateral RH. For examples of bilateral RH, see Fig. 3.

#### Vomiting

Vomiting was seen as initial symptom in six infants.

### Judicial outcome

For the 20 US infants, the judicial outcome was as follows: four children were permanently removed from home, five cases were dropped, and 11 children were returned to home after an agreement was reached with the prosecution.

A parent of one of the Norwegian children was sentenced to jail for 1.5 years in the lower court but found *not guilty* in the appeal court. Four children were temporarily removed from the family for 3.5 years before the appeal court decided that they should be repatriated, two other children were temporarily removed from their families for 0.5 and 1 year, and in the remaining two, all charges were dropped after more than a year.

## Discussion

In our combined, selected series of 28 infants where relatives denied violent shaking and diagnoses where solely based on radiological imaging showing an SDH, and in 17 cases simultaneous presence of bilateral, extensive retinal haemorrhages, all patients fulfilled the clinical and radiological diagnostic criteria for BEH. None of them had any other signs indicating injuries, such as fractures, bruises or skin swelling.

The key findings among these children were as follows:

Findings compatible with BEH:

1. A marked male preponderance (69%).
2. A large proportion was macrocephalic—81% had a head circumference larger than the 90 percentile.
3. Neuroimaging revealed radiological characteristics (CCW, SCW and IHD) that have been described/defined as typical of external hydrocephalus (BEH).

Findings compatible with SBS/AHT:

1. All included infants had chronic SDH/SDHy, in 19 infants with small amounts of acute, coagulated blood.
2. A majority—65%—of the children had bilateral retinal haemorrhages.
3. Half of the children had seizure as their first symptom.

In the following sections, we will discuss these results in detail, with special emphasis on how to avoid diagnostic pitfalls.

## Epidemiological characteristics

### Epidemiological similarities among BEH, SBS/AHT and SDH.

As discussed in “Introduction”, there are striking epidemiological similarities among SBS/AHT, BEH and SDH in infants. For all three conditions, there is a marked male preponderance [1, 22, 27–30, 68, 69, 72] and a peak appearance around 3–4 months of age [22, 27–30, 68, 69, 76]. From the literature, the rather puzzling fact emerges that *very few infants seem to have been shaken after the age of six months*. The same applies to the development of BEH and the appearance of SDH.

### Gender distribution

In the only population-based epidemiological study on BEH, the male preponderance was high—86.4% [72]. A similar overrepresentation of boys has been described in numerous other reports on BEH, see [75]. The male-to-female ratio in our material is surprisingly similar to that seen in large cohorts of infants claimed to have been subjected to vigorous shaking or abusive head trauma. Pooled together, the two studies of Adamsbaum et al. [1] and Vinchon et al. [69], with a total of 157 infants, had a marked male preponderance (73%). Considering the large number of infants included in these two studies, it appears rather unlikely that this male dominance is coincidental. In fact, this pooled male predominance in SBS/AHT infants is very close to that of the symptomatic children in the present study (69%).

### Age distribution

Also the age distribution in our population is similar to that observed in BEH populations [69, 76]; however, it matches also the age distributions of SBS/AHT [1, 69] and SDH [25, 27–29, 74]. The very early debut in all three conditions makes one wonder: is it possibly a common denominator for these conditions—and could that denominator be the birth trauma? In conjunction with what we now know about birth-related subdural blood collections, even after uncomplicated vaginal deliveries [39, 60], this early symptom debut is indeed intriguing and will be discussed in more detail below.

## Clinical and radiological findings

### Macrocephaly

Most of the included infants had large heads in the upper HC range; despite this, they were first believed to have been shaken. This is a rather surprising finding, as one would think that HC measurements in our two countries, where such measurements are routinely performed in infants, would have signalled that something abnormal was about to develop before the more dramatic symptoms, such as seizures, appeared.

Unfortunately, it is our experience that it is difficult to find exact information about HC, not to say an HC chart, in the medical records of infants assumed to have been shaken. When it comes to differentiating between a possible AHT/SBS and BEH, an HC chart is the safest tool to use, as it often will reveal an abnormally fast head growth prior to the acute worsening.

### Seizures

Half of the included infants had seizure as their first overt symptom. Contrary to what is often the assumption, seizures are quite common in children with external hydrocephalus [34, 35, 45, 51, 55, 61]. There are at least two good reasons for why external hydrocephalus complicated with SDH/SDHy should provoke seizures: increased intracranial pressure (ICP) and blood elements in the extra-cerebral fluid collections, most probably in combination.

### Retinal haemorrhages

Bilateral, extensive bleeding in several retinal layers has been regarded as a key feature of abusive head trauma [6, 7, 36, 41, 42, 53, 56]. However, RH may not be pathognomonic for abusive head traumas; they can also be seen in infants not related to abuse, e.g., in a large number of healthy newborns [37, 50, 70], in infants with “macrocephaly” [55, 64], after “high-risk” deliveries [58], following acute life-threatening events [5], and after cardiopulmonary resuscitation [33, 54, 57]. RHs have also been documented in premature infants; contrary to the rapid resolution of the bleeding one usually sees in most newborns, the bleeding in preterms tend to be long lasting [18]. Four of our included children were prematurely born.

What are then the mechanisms behind the intraocular bleeding in “non-shaken” infants? The most likely explanation seems to be the transmission of an increased ICP through the optic nerve sheath to the intraocular compartment, causing Terson’s syndrome [12]. In a patient cohort comprising much older children (three years or older), it was demonstrated that increased ICP alone could cause retinal haemorrhage [8], however not as extensive as in the included infants. The optic nerve sheath is much shorter in infants than in older children; consequently, one would expect an increased ICP to be conveyed more easily to the eye and cause retinal bleeding in infants.

### Vomiting

Vomiting and regurgitation are commonly reported phenomena in BEH [46, 73].

## Neuroimaging

### Subdural haematomas and hygromas

As the neuroimaging studies in the included cases most often were performed in close relation to the initial symptoms, one would expect these early CT and/or MRI scans to show features compatible with an *acute* intracranial traumatic haemorrhagic event, such as an acute subdural haematoma (ASDH), and that this haematoma had caused a compression of the ipsilateral brain and ventricle with a midline shift when unilateral, and compression of both hemispheres and lateral ventricles if bilateral, as one usually sees in traumatic ASDH. On the contrary, this infants’ neuroimaging failed to reveal features of an acutely acquired haematoma caused by external traumatic forces. Acute, coagulated blood was *not* predominant; the extra-cerebral fluid seemed more compatible with *chronic* SDH (CSDH) or SDHy, sometimes with scattered small volumes of coagulated blood. Moreover, the subdural fluid collections did in general not exert any mass effect on the cortical surface or the lateral ventricles, the former was not flattened, and the latter mostly appeared to be of normal size or even moderately enlarged. Unilateral extra-axial fluid collections did not cause midline shift or compression of the ipsilateral ventricle, and bilateral CSDH did not cause any marked cortical flattening or ventricular compression. These observations indicate that the increased intracranial pressure is evenly distributed between the extra-cerebral fluid compartment, and the ventricles and the lack of compression of the underlying brain may indicate these chronic fluid collections merely occupy an extra-axial fluid compartment that is preexisting due to the external hydrocephalus.

In short, the neuroimaging in these infants appeared to be more compatible with BEH complicated by CSDH/SDHy, a phenomenon that is well-known in infants with BEH [3, 4, 21, 23, 26, 31, 35, 44, 45, 47, 48, 52, 55, 59, 67, 69], than an acute traumatic event. CSDHs, rather than acute haemorrhage, may also be the result of previous injuries. All these features overlap with BEH but could possibly also represent previous episodes of abuse.

There are several possible mechanisms for this predisposition for SDH in BEH infants. For the last decade, it has been known that large proportions of newborns have subdural blood [60]. It is possible that this subdural blood gradually develops into larger haematomas, as there are growth factors in old haematomas that induce neo-vascularization with pathological vessels that bleed easily [19, 62, 71] and other factors in the SDH that disturb normal coagulation [32, 49]. A recent article discusses the possibility that this birth-related subdural blood in itself may cause development of BEH [77].

There is also another possible explanation of this predisposition: the stretched bridging veins in BEH. It is a common intraoperative observation during craniotomies that even minute and careful manipulation of normal bridging veins may cause oozing of blood from where these veins enter the dura. With an increased amount of extra-cerebral fluid, these veins may leak blood spontaneously at the entry points just because they are stretched.

## How can we improve the diagnostic safety in SBS/AHT in order to avoid BEH being misdiagnosed as a result of abuse?

It follows from above that BEH complicated by SDH/SDHy possibly or even probably can be, and has been, misdiagnosed as SBS/AHT. Based on our personal experience and the results of the present study, we suggest the following measures to improve diagnostic safety:

1. All physicians that act as medical experts in infant abuse cases should be informed about BEH as a possible differential diagnosis to SBS/AHT. They should, however, not only be informed, they should also be *trained* in recognising clinical and radiological features of BEH.
2. Nearly all infants that have been diagnosed as abused, have been so by colleagues without *clinical* experience in diagnosing and managing infants with acute intracranial conditions. Nearly all our included infants were diagnosed as abused by CAPs or specialists in forensic medicine; very rarely are *clinicians* with relevant clinical experience involved. In our opinion, leaving the diagnostic work in such cases to colleagues without clinical experience with similar conditions will represent a continued risk of misdiagnosing BEH as SBS/AHT. Therefore, clinicians experienced in paediatric neurology and neurosurgery, including hydrocephalus and traumatic head injuries, should be present in all expert teams. We believe this measure will reduce the risk of missing out BEH as a possible alternative to abuse diagnoses.
3. Increased head circumference (HC) or a rapidly increasing HC is the most important clinical feature when diagnosing BEH. Therefore, experts in such cases should ensure that they have reliable information about the HC development from birth, either in a HC chart or as numerical measurements in the medical records before they conclude.

## The scientific solidity of the medical evidence—the “triad”—behind the SBS/AHT diagnosis.

Literature reviews have concluded that it is difficult to find scientific evidence above level 3 for a causal relationship between the triad and violent shaking/abuse [13]. More recently, a similar review based on thousands of articles came to the following conclusions: “There is limited scientific evidence that the triad and therefore its components can be associated with traumatic shaking (low-quality evidence). There is insufficient scientific evidence on which to assess the diagnostic accuracy of the triad in identifying traumatic shaking (very low-quality evidence)” [15, 63].

Despite a vast amount of publications on SBS/AHT, there appears to be no studies based on *observed* shaking, but two studies in the literature are based on confessed shaking [1, 68]. These confessions, however, came weeks to months after the diagnosis had been made, and they were obtained during police or judicial investigations. Confessions obtained under such circumstances are known to be associated with uncertainties [24]. Thus, the diagnosis of SBS/AHT appears to be based on weak medical evidence; in Sweden, the triad is no longer viewed as proof of shaking [15]. A recent study on 36 infants, where violent shaking had been observed or admitted when the baby was hospitalised, failed to show the typical findings of the triad, with the exception of two babies with preexisting vulnerability, who had acute extra-axial haematoma, but no other findings [66].

The important issue is that a diagnosis or conviction cannot be based on the triad alone. SBS/AHT is usually not an isolated event, but a behavioural pattern. Infants typically present after a severe episode, or when accumulated injuries following multiple episodes reach a critical point. A triad may occur as caused by shaking or as independent of shaking. It seems difficult either to prove or to disprove SBS/AHT based on medical evidence, i.e., clinical or radiological findings.

## Weakness of this study

We are aware that the selection of infants presented in this article is heavily biased, as all children were selected based on self-referral of parents seeking medical expertise for court proceedings for a suspicion of having been violently shaken. They are therefore neither representative for all infants with BEH nor all infants who were victims to abuse; the clinical and radiological features described here may only be found in a small proportion of BEH infants. Our aim was not to provide population-based analyses, but to raise awareness about a potentially devastating pitfall in the diagnosis of AHT/SBS.

## Conclusions

We have described a cohort of children that were suspected of being victims of violent shaking, but who met the diagnostic criteria of BEH. We consider BEH as an important differential diagnosis when children are diagnosed with a single or all components of the triad that has been used to describe SBS/AHT, especially since solid data indicate that a diagnosis of SBS/AHT should not be made solely from a clinical triad. To avoid missing out BEH as a differential diagnosis, we suggest that paediatric neuro-*clinicians* always are engaged in the expert team. As macrocephaly is the most important clinical symptom in BEH, information on the development of head circumference should always be available.

## Abbreviations

AHT: Abusive head trauma
ASDH: Acute subdural haematoma
BEH: Benign external hydrocephalus
BESS: Benign enlargement of the subarachnoid spaces
CSF: Cerebrospinal fluid
HC: Head circumference
RH: Retinal haemorrhages
SBS: Shaken baby syndrome
SDH: Subdural haematoma
SDHy: Subdural hygroma
